# Traumatic Life Events and Association With Depression, Anxiety, and Somatization Symptoms in Female Refugees

**DOI:** 10.1001/jamanetworkopen.2023.24511

**Published:** 2023-07-20

**Authors:** James Kenneth Moran, Jenny Jesuthasan, Inga Schalinski, Christine Kurmeyer, Sabine Oertelt-Prigione, Ingar Abels, Ulrich Stangier, Annabelle Starck, Jana Gutermann, Ulrike Zier, Anja Wollny, Knejinja Richter, Antje Krüger, Meryam Schouler-Ocak

**Affiliations:** 1 Department of Psychiatry and Psychotherapy, Multisensory Integration Lab, Charité Universitätsmedizin, St Hedwig Hospital, Berlin, Germany; 2 Psychiatric University Clinic Charité, St Hedwig Hospital, Berlin, Germany; 3 Universität der Bundeswehr München, Department of Human Sciences, Institute of Psychology, Munich, Germany; 4 Office of the Equal Opportunities Officer, Charité–Universitätsmedizin, Berlin, Germany; 5 Department of Primary and Community Care, Radboud Institute for Health Sciences, Radboud University Medical Center, Nijmegen, the Netherlands; 6 AG 10 Sex- and Gender-Sensitive Medicine, Medical Faculty OWL, University of Bielefeld, Bielefeld, Germany; 7 Clinical Psychology and Psychotherapy, Department of Psychology, Goethe-University Frankfurt, Frankfurt, Germany; 8 Institute of Occupational, Social, and Environmental Medicine, University Medical Center of the Johannes Gutenberg University, Mainz, Germany; 9 Now with Ministry of Science and Health of Rhineland-Palatinate, Mainz, Germany; 10 Institute of General Practice, University Medical Center Rostock, Rostock, Germany; 11 CuraMed Tagesklinik Nürnberg, Nuremberg, Germany; 12 Technische Hochschule Nürnberg, Nuremberg, Germany

## Abstract

**Importance:**

Different types of traumatic life events have varying impacts on symptoms of depression, anxiety, and somatization. For women from areas of the world experiencing war and humanitarian crises, who have experienced cumulative trauma exposure during war and forced migration, it is not known whether cumulative trauma or particular events have the greatest impact on symptoms.

**Objective:**

To examine which traumatic life events are associated with depression, anxiety, and somatization symptoms, compared with the cumulative amount, in a sample of female refugees.

**Design, Setting, and Participants:**

For this cross-sectional study, data were collected in 2016 as a part of The Study on Female Refugees. The current analysis was conducted in 2022 to 2023. This multicenter study covered 5 provinces in Germany. Participants were recruited at reception centers for refugees. Women volunteered to participate and to be interviewed after information seminars at the different centers.

**Exposure:**

Traumatic life events experienced by refugees from areas of the world experiencing war and humanitarian crises.

**Main Outcomes and Measures:**

Demographic variables (age, country of origin, religion, education, relationship status, and children), traumatic and adverse life events, and self-reported depression, anxiety, and somatization symptoms were measured. Random forest regressions simultaneously examined the importance of these variables on symptom scores. Follow-up exploratory mediation models tested potential associative pathways between the identified variables of importance.

**Results:**

For the final sample of 620 refugee women (mean [SD] age, 32.34 [10.35] years), family violence was most associated with depression (mean [SD] variable of importance [VIM], 2.93 [0.09]), anxiety (mean [SD] VIM, 4.15 [0.11]), and somatization (mean [SD] VIM, 3.99 [0.15]), even though it was less common than other traumatic experiences, including war, accidents, hunger, or lack of housing. Other factors, such as childhood sexual abuse, injury, near-death experiences, and lack of access to health care, were also important. Follow-up analyses showed partial mediation effects between these factors in their association with symptoms, supporting the unique importance of family violence in understanding mental health.

**Conclusions and Relevance:**

The findings of this cross-sectional study of refugee women who experienced multiple severe traumas related to war in their home countries and danger encountered during their migration suggest that family violence was key to their current mental health problems. Culturally sensitive assessment and treatment need to place special emphasis on these family dynamics.

## Key Points

**Question:**

Which traumatic life events are associated with current depression, anxiety, and somatization symptoms in female refugees seeking psychological help?

**Findings:**

In this cross-sectional study of 620 female refugees, by use of random forest regression, family violence had the highest scores associated with depression, anxiety, and somatization, beyond cumulative trauma exposure. These scores were higher than those for more frequently reported traumatic events, such as war, accident, lack of housing, hunger, and near death.

**Meaning:**

These findings suggest that even for women with a history of multiple traumatic experiences, understanding the impact and likelihood of family violence is vital in diagnostic assessments and informing treatment strategies.

## Teaser

This cross-sectional study examines which traumatic life events are associated with depression, anxiety, and somatization symptoms, beyond cumulative trauma exposure, in a sample of female refugees.

## Introduction

The displacement of people from their countries through war and conflict, owing to direct threats to individual lives and famines, has increased continually in the last decade, with more than 80 million displaced persons recorded in 2020.^1^ Germany experienced a major influx of refugees and asylum seekers beginning in 2015,^2^ primarily owing to the Syrian civil war, as well as other conflicts and humanitarian crises in the Middle East and North Africa. At the time of this writing in 2023, a new wave of refugees is fleeing war in Ukraine.^3^ Many refugees experience repeated traumatic events, physical and sexual violence, torture and imprisonment, and witnessing the death of loved ones, as well as frequent interpersonal violence, including emotional, physical, and sexual abuse.^4^ These experiences increase the risk of a wide variety of mental health issues, including posttraumatic stress disorder (PTSD), depression, anxiety, and somatization.^5,6^ Given the scale of this epidemiological trend, it is imperative to better map the occurrence of mental health problems and their exposure-based antecedents to evaluate risks in diagnostic assessments and optimize treatment interventions.

A challenge of understanding the exposure-based antecedents of mental illness in refugee populations is finding a way to group the data. A dose-response relationship can be demonstrated across different clusters of psychopathology symptoms by summing different types of traumatic life events. This building block effect, as seen in PTSD,^7,8^ where traumatic experiences are part of the definition and cause of the disorder, has also been shown to be relevant in depression and anxiety^9,10,11^ and in somatization.^12^ However, this simplification of the data has the disadvantage of missing differences in the impacts of the types of traumatic events. Other studies have focused on one type of life event, such as studies showing that intimate partner violence is associated with depression.^13^ Although these findings are valid within the scope of the respective studies, there is a disadvantage in that the broader context of the individual’s life is not accounted for. For example, within refugee populations, somatization^14,15,16^ is associated with previous traumatic experiences,^17^ childhood sexual abuse (CSA) in particular.^18,19,20^ However, we do not know whether this is a primary factor, or if it is instead associated with other later traumatic events, such as domestic violence or personal injury.

One way of reducing the complexity of the picture is by clustering different types of traumatic events. For example, in a study^12^ of depression, anxiety, and somatization, trauma was categorized according to human rights abuses, human needs, separation, and traumatic loss. Following this approach, many studies^21,22,23,24^ make a distinction between assaultive, human-made violence, natural disasters, accidents, or witnessing violence in others. In the present study, we use a different, more data-driven approach to simultaneously examine individual traumatic experiences and their cumulative impact on symptoms. We interviewed a large group of female refugees, the majority of whom were fleeing war in Syria and Afghanistan; however, there were also others from North Africa and other Middle Eastern countries. As such, these women experienced extremes of human suffering, including war, torture, displacement, and sexual and gender-based violence.^25^ Our question was whether their symptom levels were primarily associated with the cumulative impact of multiple trauma, or whether particular traumatic experiences had a greater impact on their current state.^26,27^ We do not know which events have the greatest impact on their present levels of depression, anxiety, or somatization, beyond the cumulative impact of their traumatic experiences. Furthermore, although these symptoms cluster together,^12,27^ it is possible that the different expression of symptoms is associated with different types of events. However, this is important to better understand refugees, target interventions, and, ultimately, help with integration in the host society.

The present study uses random forest regression to comprehensively evaluate factors associated with symptoms of anxiety, depression, and somatization in refugee women with various forms of risks and cultural backgrounds.^28^ This enables a fine-grained analysis of the impact of individual events possible, even in smaller sample sizes, by allowing the simultaneous consideration of risks in association with symptoms of mental health.^29^ This approach has already been applied to PTSD and aggression.^30,31^ By identifying the most important variables accounting for symptoms, we further test potential mediations between variables of importance (VIMs).

## Methods

### Recruitment

This cross-sectional study was a joint project (The Study on Female Refugees) conducted in 2016 with 5 locations in 5 provinces in Germany: Berlin (the capital city), Mainz in Rhineland-Palatinate, Nuremberg in Bavaria, Rostock in Mecklenburg–Western Pomerania, and Frankfurt in Hesse. All project partners sought and obtained ethical approval within their institution of reference (university or region, depending on the regional law). All procedures complied with the declaration of Helsinki.^32^ This study followed the Strengthening the Reporting of Observational Studies in Epidemiology (STROBE) reporting guideline.

Participants were recruited at the reception centers for refugees in collaboration with facility management. The project was introduced via a 1.5-hour information event presented by native speakers of Arabic, Dari or Farsi, Somali, and Tigrinya, followed up by flyers in the respective languages. Women (aged ≥18 years) who were interested were interviewed privately at reception facilities after at least a 1-day interval. Informed consent was obtained in either written form or in oral form, if the participant was illiterate. Waivers of consent were not required. Interviews were conducted in the participants’ native language by trained women. The interviewers’ qualifications and degrees ranged from student status to doctorate degree. They received a 1.5-day training on interviewing traumatized refugees and mental health, dealing with refugees showing signs of distress, and referral contacts. They were briefed on the protection of their own mental health, mandatory supervision appointments, and further counseling, if they noticed any symptoms of distress on their own.

The women could fill in the questionnaire themselves if they were literate, with support for the open questions. If they were illiterate, the interviewer read the questions and filled in the form themselves. A single follow-up question was asked if the participants left the space blank. Further questioning was avoided to minimize the possibility of retraumatization.

### Measures

The measures were part of a questionnaire battery.^4,11^ We used the Harvard Trauma Questionnaire (HTQ)^33,34^ and the Posttraumatic Diagnostic Scale to assess both witnessed and personally experienced lifetime traumatic experiences. Depression and anxiety were assessed with their respective subscales of the Hopkins Symptom Checklist (HSCL-25).^35^ Somatization was assessed using the Symptom Checklist–90 Revised (SCL-90-R) questionnaire, somatization subscales.^36^ See Starck et al^11^ for details of psychometric properties. In addition, demographic details were collected. The original qualitative data on these aspects were simplified for compatibility with the random forest procedure (see the Statistical Analysis subsection). These included marital status (in a relationship or alone), country of origin, education level (no school, school, or tertiary education), religion (Islam, Christian, or other), children (yes or no), and age.

### Statistical Analysis

The current analysis was conducted in 2022 to 2023. To test the associations of current symptoms (depression, anxiety, and somatization) with traumatic life events, together with demographic factors, the machine learning technique conditioned random forest regression was used.^30^ Conventional multiple regression would not be appropriate for the analysis, because there are many variables, even relative to our large data set, with high multicollinearity. A random forest regression does not require variables to be normally distributed; the number of variables can, in principle, exceed the number of data points, and the decision tree structure can account for interactions between variables. Thus, the power of our sample size is adequate to this form of analysis. The relative importance of each variable can be estimated via random permutation of each variable and testing the resultant degradation of model fit. Permutation of important independent variables will reduce model fit, whereas unimportant variables will have little effect.

For this analysis, we used the cforest package in R statistical software version 4.1.2 (R Project for Statistical Computing).^37^ This has been used previously^38^ and has further refinements to remove biases from the importance resulting from number of categories, mean values, range, or variance in independent variables. The script was based on the procedure described by Schalinski et al.^39^ The training and testing models used 10 repetitions of 10-fold cross-validation. Nine of the 10 sets are used to generate the model, which is then tested on the 10th set. This is repeated 10 times, so that each set tests the validity of the model. This whole process is repeated 10 times on different random sets of the data. The results provide 95% CIs and *P* value estimates for each independent variable. For each of the 3 analyses, *P* values were adjusted with the Benjamini-Hochberg correction. Three separate analyses each were performed for HSCL Depression subscale, the HSCL Anxiety subscale, and the SCL-90-R Somatization subscale. The independent variables were demographic factors (ie, age, education, relationship status, children, country, and religion), HTQ traumatic life experiences, and cumulative scores of HTQ traumatic life experiences.

Although the algorithm is robust to collinearity in independent variables, it is not possible to directly test associations between independent variables as one would with standard parametric statistical analyses. Therefore, once VIMs were defined, we performed exploratory follow-up analyses examining the associations between these independent variables. Where 2 categories of traumatic experiences are associated with depression, it is possible that the more proximate one mediates the less proximate one. We tested this with mediation analyses of VIMs via the Mediation package in R.^40^ Mediation effects were tested with bootstrapping (1000 samples), estimating explained variance over the indirect path, with 95% CIs. To ensure adequate power, we checked the crosstabulation of risk factors of interest to ensure a minimum of responses in each cell; the smallest cell across all analyses was 27.

## Results

### Descriptive Statistics

In all, we recruited 663 women: 116 from Bavaria, 257 from the city of Berlin, 98 from Hesse, 105 from Mecklenburg-Western Pomerania, and 87 from Rhineland-Palatinate. The sample size was reduced from 663 to 620 because the analysis is sensitive to missing data in outcome variables; thus, 620 women (mean [SD] age, 32.34 [10.35] years) participated in the study. A large majority of women came from war-afflicted countries in the Middle East, with Syria (278 women [45.2%]), Afghanistan (148 women [24.1%]), and Iraq (71 women [11.5%]) as the most frequent countries of origin (Table 1). The majority of women (451 women [72.7%]) were in a relationship, had children (497 women [80.2%]), and had some form of education, with 105 (16.9%) having not gone to school. Islam was the most common religion (505 women [81.5%]). Participants experienced a mean (SD) of 5.68 (4.05) traumatic experiences, including family violence (116 women [18.7%]), war (337 women [54.3%]), accidents (322 [51.9%]), lack of housing (318 women [51.3%]), and hunger (285 women [45.9%]) (Table 2).

**Table 1.** Demographic and Outcome Variables Entered Into the Random Forest Analyses

Variable	Participants, No. (%)
Demographic variables	
Relationship status (n = 620)	
In a relationship	451 (72.7)
Alone	169 (27.3)
Education (n = 615)	
No school	105 (16.9)
School	448 (72.5)
Tertiary	67 (10.8)
Religion (n = 620)	
Islam	505 (81.5)
Christian	64 (10.3)
Other^a^	43 (6.9)
None	8 (1.3)
Children (n = 620)	
Yes	497 (80.2)
No	123 (19.8)
Country of origin (n = 615)	
Afghanistan	148 (24.1)
Eritrea	42 (6.8)
Ethiopia	13 (2.1)
Iran	36 (5.9)
Iraq	71 (11.5)
Lebanon	4 (0.7)
Saudi Arabia	3 (0.5)
Somalia	17 (2.8)
Syria	278 (45.2)
Ukraine	1 (0.2)
United Arab Emirates	2 (0.3)
Continuous variables, mean (SD) [range]^b^	
Age, y	32.34 (10.35) [18.00-69.00]
Symptom Checklist–90 Revised somatization subscale	2.18 (0.92) [1.00-5.00]
HSCL Anxiety	2.22 (0.80) [1.00-4.10]
HSCL Depression	2.27 (0.72) [1.00-3.87]
Harvard Trauma Questionnaire	5.68 (4.05) [0.00-19.00]

Abbreviation: HSCL, Hopkins Symptom Checklist.

^a^ Includes Hinduism, none, private, Yazidi, and Zoroastrian.

^b^ Scores for somatization, HSCL Anxiety, and HSCL Depression and number of traumatic events experienced, as measured by the Harvard Trauma Questionnaire, are reported for the entire sample of 620 women.

**Table 2.** Experience of Different Traumatic Events

Traumatic life events^a^	Participants, No. (%) (N = 620)
War (self)	337 (54.3)
Accident	322 (51.9)
Lack of housing (self)	318 (51.3)
Hunger (self)	285 (45.9)
Near death (self)	252 (40.7)
Health problem and no access to health care	222 (35.8)
Separation (self)	193 (31.1)
Natural disaster	171 (27.7)
Sudden death	164 (26.4)
Murder own family	163 (26.2)
Violence (self)	134 (21.7)
Family violence (self)	116 (18.7)
Isolation (self)	94 (15.1)
Torture (self)	85 (13.7)
Kidnapping (self)	84 (13.5)
Injury (self)	79 (12.9)
Prison (self)	77 (12.5)
Childhood sexual abuse (self)	75 (12.1)
Murder stranger (self)	70 (11.3)
Life-threatening illness	69 (11.2)
Violence (witness)	63 (10.2)
Sexual violence stranger	53 (8.5)
Torture (witness)	51 (8.2)
Family violence (witness)	41 (6.7)
Sexual violence family	28 (4.6)

^a^ Events were measured by the Harvard Trauma Questionnaire and are shown in descending order of prevalence. Self refers to the type of confrontation with the traumatic event, whereas witness refers to the witnessing of a traumatic event.

### Random Forest Analyses

**Figure 1.**
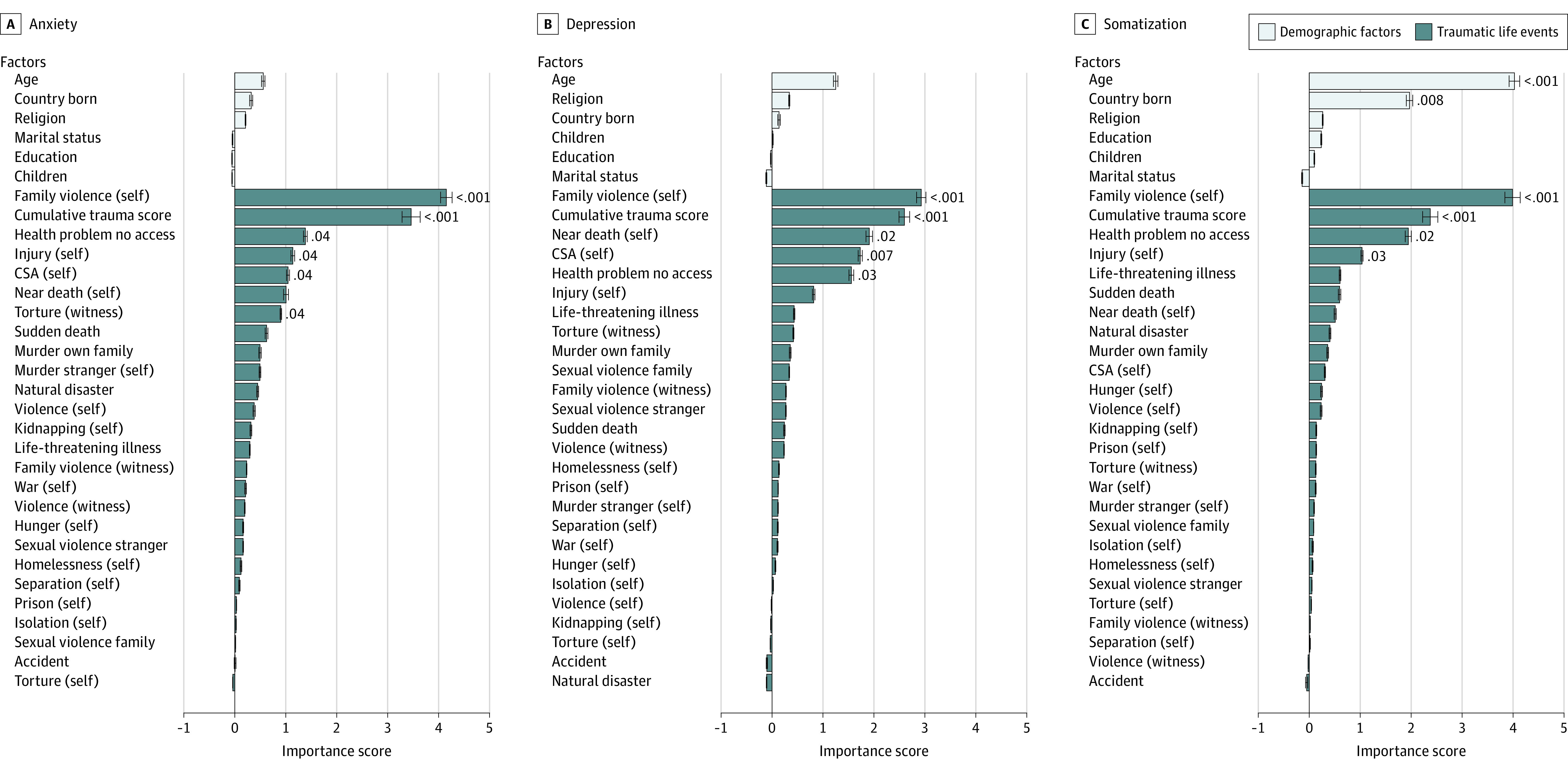
Random Forest Regression Analysis of Factors Associated With Current Psychopathology Anxiety (A), depression (B), and somatization (C) symptoms were measured by the Hopkins Symptom Checklist Depression Subscale. Demographic factors are shown by light bars. Traumatic life events are shown by dark bars. Otherwise, the size of their contribution to the model is ordered from top to bottom descending. Benjamini-Hochberg–corrected *P* values are shown for significant factors after permutation test of the model (5000 permutations). Confidence intervals are within 1 SD of the permutations. CSA indicates childhood sexual abuse.

The random-forest regressions for depression, anxiety, and somatization yielded both common and differential factors associated with traumatic life events and demographic characteristics (Figure 1). Family violence was the largest VIM across anxiety (mean [SD] VIM, 4.15 [0.11]), depression (mean [SD] VIM, 2.93 [0.09]), and somatization (mean [SD] VIM, 3.99 [0.15]) (Benjamini-Hochberg–corrected *P *< .001 for all). This was followed by the cumulative trauma score for all 3 symptom types (mean [SD] VIMs, 3.46 [0.18] for anxiety, 2.60 [0.11] for depression, and 2.37 [0.15] for somatization; Benjamini-Hochberg–corrected *P *< .001 for all). Lack of access to health care was associated with symptoms for all 3 symptom types (mean [SD] VIMs, 1.38 [0.04] for anxiety [Benjamini-Hochberg–corrected *P* = .04], 1.56 [0.05] for depression [Benjamini-Hochberg–corrected *P* = .03], and 1.94 [0.06] for somatization [Benjamini-Hochberg–corrected *P* = .02]). After this, there were differences between scores. CSA was associated with anxiety (mean [SD] VIM, 1.05 [0.03]; Benjamini-Hochberg–corrected *P* = .04) and depression (mean [SD] VIM, 1.73 [0.04]; Benjamini-Hochberg–corrected *P* = .007), whereas somatization was not (mean [SD] VIM, 0.31 [0.01]; Benjamini-Hochberg–corrected *P* = .28). Injury was associated with anxiety (mean [SD] VIM, 1.14 [0.04]; Benjamini-Hochberg–corrected *P *= .04) and somatization (mean [SD] VIM, 1.04 [0.02]; Benjamini-Hochberg–corrected *P *= .03), but not depression (mean [SD] VIM, 0.82 [0.02]; Benjamini-Hochberg–corrected *P *= .07). Near death was associated with depression (mean [SD] VIM, 1.91 [0.06]; Benjamini-Hochberg–corrected *P *= .01). Age (mean [SD] VIM, 4.03 [0.10]; Benjamini-Hochberg–corrected *P *< .001) and country of origin (mean [SD] VIM, 1.97 [0.06]; Benjamini-Hochberg–corrected *P *< .001) were associated with higher levels of somatization. The meaning of the difference in country of origin is difficult to interpret statistically, because the numbers for different countries range so radically from 1 to 278.

### Follow-Up Analyses

**Figure 2.**
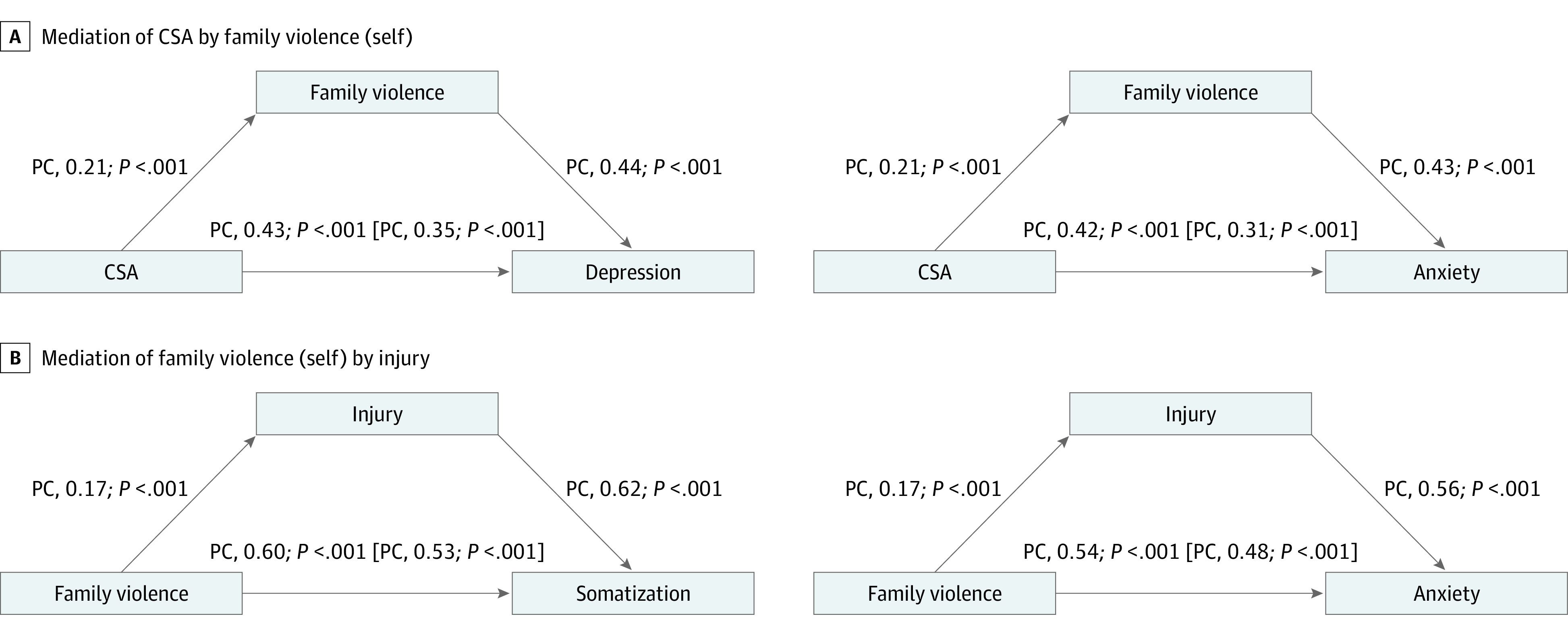
Mediation Models Assessing Possible Mediation of Associations Between Variables of Importance in the Random Forest Regression Panel A depicts whether the association of childhood sexual abuse (CSA) with depression and anxiety was mediated by family violence (self). Both independent variables contributed main effects to the respective symptoms, with a partial mediation effect (in square brackets). Panel B shows that the association with family violence (self) was mediated by high levels of injury, explaining variance in both anxiety and somatization. Both independent variables contributed main effects to the respective symptoms, with a partial mediation effect (in square brackets). PC indicates path coefficient.

The identified VIMs show the importance of family violence across depression, anxiety, and somatization. However, family violence could have complex associations with other VIMs. Therefore, we tested potential mediation models. For both depression and anxiety, both CSA and family violence were VIMs. If CSA is a factor associated with adult relationship problems, its association with depression or anxiety and depression could be mediated by more recent family violence. However, both factors were independently associated with depression and anxiety, with small partial mediation effects (Figure 2A). The indirect path accounted for 8.0% (95% CI, 3.2%-13.0%; *P* < .001) of variance in depression and 10.8% (95% CI, 4.5%-10.8%; *P* < .001) of variance in anxiety. Both somatization and anxiety were associated with family violence and physical injury (Figure 2A). The effect of family violence on symptom scores could be indirectly associated with physical injury. However, the models suggested independent contributions to symptom ratings, with small partial mediation effects. The indirect path accounted for 6.3% (95% CI, 2.5%-11.0%; *P* < .001) of variance in anxiety and 7.9% (95% CI, 3.2%-14.0%; *P* < .001) of variance in somatization.

## Discussion

The aim of this cross-sectional study was to simultaneously examine individual types of traumatic events, as well as the cumulative impact of trauma, to see which have the greatest impact on current depression, anxiety, and somatization symptoms in a group of refugee women. This study supports previous findings of the importance of the cumulative impact of traumatic life events for present symptoms for depression and anxiety^9,10,11^ and for somatization.^12^ Beyond the cumulative amount of trauma exposure, the present findings indicate that family violence experienced by refugee women had the greatest contribution across all forms of symptoms, emphasizing the central importance of interpersonal trauma and insecurity. Finally, for anxiety, depression, and somatization, there was variance in the contributors to the different psychological symptom profiles, discussed individually in the following sections.

Previous studies^21,23,24,41^ suggest that interpersonal events have a greater impact on psychopathology than noninterpersonal events. For example, Haldane et al,^41^ by differentiating between interpersonal and noninterpersonal trauma, found that women’s PTSD and anxiety symptoms were particularly associated with interpersonal traumatic events. Our analysis is novel in that it enabled a more detailed examination of individual types of trauma, rather than broader categories of traumatic experience, to show the importance of family violence. This is in spite of the fact that family violence was a less common form of trauma in our cohort (18.7% of women) in comparison to many other more frequent traumas, including war (54.3%), accidents (51.9%), lack of housing (51.3%), and hunger (45.9%). Indeed, the model suggested that family violence has a contribution greater than the cumulative impact of all traumatic events across depression, anxiety, and somatization.

Both depression and anxiety showed many commonalities in contributing factors, including CSA, lack of health care access, and near death experiences. Anxiety scores were also associated with witnessing torture. For somatization, illness without access to health care and injury were important. The types of family violence encompassed include chronic abuse from a spouse, as well as individual attacks by family members. It is possible that physical injury is a direct follow-on effect of family violence; however, mediation analyses found partial indirect effects, in which family violence contributed directly to anxiety and depression, independently of physical injury. Similarly, our follow-up analyses of family violence as a potential mediating factor associated with CSA, showed that both family violence and CSA were independently associated with symptoms.

This study provides a comprehensive picture of the impact of a wide range of life events and shows that family violence has a great impact on symptoms overall. It is necessary to follow-up these findings further using longitudinal study designs and cross-lag analyses, which would help identify stable associations of exposure risks with psychopathology. Family violence is a potentially more immediate part of the current postmigration existence. There is a connection between experiences of war and violence in the home.^42^ For example, a study^43^ of Iraqi refugees showed that recent experiences of domestic violence were associated with the psychopathology of the male partner (PTSD and depression), as well as cultural attitudes. Other factors, such as substance abuse, destabilization of customs, and breakdown of social and familial bonds, could also contribute to a greater impact of family-related violence.^44^

Understanding the centrality of family violence is important in treating vulnerable minority groups, because they already have problems accessing adequate health care in their host countries.^45^ Despite limited access to health infrastructure, refugees have legal rights to particular forms of care, including gynecologists and other obstetric health professionals, such as midwives, as well as inpatient obstetrics and pediatric centers. This could be a means of reaching these women, providing direct emotional support, as well as referring them to counseling, shelter, or legal aid. Training initiatives for social workers in the shelters and health care professionals to deal with the taboo aspect of domestic violence to facilitate such a protective framework are important.^46,47^

### Limitations

This study has limitations that should be mentioned. Our approach gave a broad picture of questionnaire-based symptoms of a large population sample, rather than clinical diagnoses. Additional assessment of PTSD symptoms might have provided a more rounded picture of the symptom profile, since many participants had high levels of trauma experiences (mean, 5.68 experiences), which is factor associated with PTSD,^7^ and anxiety, depression, and somatization form part of this constellation of symptoms.^48^ Levels of medication, particularly psychopharmaceutical help, would also enrich the picture of the data. In addition, our recruitment method could not ensure a randomized sample, as participation was driven by the interest of female volunteers, limiting the generalizability of our findings beyond those seeking help.

## Conclusions

The present study provides insights into the most important factors associated with increase risk of trauma in a large help-seeking sample of female refugees. Beyond the cumulative amount of trauma, exposure to family violence appears to be the key factor associated with risk of current symptoms of anxiety, depression, and somatization. Thus, the diagnostic assessment of exposure to trauma types may be relevant to identify women with increased risk for psychopathology, assign specific interventions addressing family violence, and, thereby, optimize treatment outcomes.

## Notes

Supplement.
Data Sharing Statement

## References

[1] United Nations Refugee Agency. UNHCR global trends: forced displacement in 2020. Accessed April 1, 2022. https://www.unhcr.org/flagship-reports/globaltrends/

[2] Bäärnhielm S, Laban K, Schouler-Ocak M, Rousseau C, Kirmayer LJ. Mental health for refugees, asylum seekers and displaced persons: a call for a humanitarian agenda. Transcult Psychiatry. 2017;54(5-6):565-574. doi:10.1177/1363461517747095 29226788

[3] Brücker H, Goßner L, Hauptmann A, . Die Folgen Des Ukraine-Kriegs Für Migration Und Integration: Eine Erste Einschätzung. Institut für Arbeitsmarkt- und Berufsforschung. Institute for Employment Research; 2022. Accessed April 1, 2022. https://ideas.repec.org/p/iab/iabfob/202202.html

[4] Jesuthasan J, Sönmez E, Abels I, ; Female Refugee Study (FRS) Investigators. Near-death experiences, attacks by family members, and absence of health care in their home countries affect the quality of life of refugee women in Germany: a multi-region, cross-sectional, gender-sensitive study. BMC Med. 2018;16(1):15. doi:10.1186/s12916-017-1003-5 29391012 PMC5793395

[5] Henkelmann JR, de Best S, Deckers C, . Anxiety, depression and post-traumatic stress disorder in refugees resettling in high-income countries: systematic review and meta-analysis. BJPsych Open. 2020;6(4):e68. doi:10.1192/bjo.2020.54 32611475 PMC7443922

[6] Sambucini D, Aceto P, Begotaraj E, Lai C. Efficacy of psychological interventions on depression anxiety and somatization in migrants: a meta-analysis. J Immigr Minor Health. 2020;22(6):1320-1346. doi:10.1007/s10903-020-01055-w 32712851 PMC7683473

[7] Neuner F, Schauer M, Karunakara U, Klaschik C, Robert C, Elbert T. Psychological trauma and evidence for enhanced vulnerability for posttraumatic stress disorder through previous trauma among West Nile refugees. BMC Psychiatry. 2004;4(1):34. doi:10.1186/1471-244X-4-34 15504233 PMC529265

[8] Wilker S, Pfeiffer A, Kolassa S, Koslowski D, Elbert T, Kolassa IT. How to quantify exposure to traumatic stress? reliability and predictive validity of measures for cumulative trauma exposure in a post-conflict population. Eur J Psychotraumatol. 2015;6(1):28306. doi:10.3402/ejpt.v6.28306 26589255 PMC4654773

[9] Oppedal B, Özer S, Şirin SR. Traumatic events, social support and depression: Syrian refugee children in Turkish camps. Vulnerable Child Youth Stud. 2018;13(1):46-59. doi:10.1080/17450128.2017.1372653

[10] Price M, van Stolk-Cooke K. Examination of the interrelations between the factors of PTSD, major depression, and generalized anxiety disorder in a heterogeneous trauma-exposed sample using DSM 5 criteria. J Affect Disord. 2015;186:149-155. doi:10.1016/j.jad.2015.06.012 26241663

[11] Starck A, Gutermann J, Schouler-Ocak M, Jesuthasan J, Bongard S, Stangier U. The relationship of acculturation, traumatic events and depression in female refugees. Front Psychol. 2020;11:906. doi:10.3389/fpsyg.2020.00906 32528358 PMC7247808

[12] Jongedijk RA, Eising DD, van der Aa N, Kleber RJ, Boelen PA. Severity profiles of posttraumatic stress, depression, anxiety, and somatization symptoms in treatment seeking traumatized refugees. J Affect Disord. 2020;266:71-81. doi:10.1016/j.jad.2020.01.077 32056948

[13] Devries KM, Mak JY, Bacchus LJ, . Intimate partner violence and incident depressive symptoms and suicide attempts: a systematic review of longitudinal studies. PLoS Med. 2013;10(5):e1001439. doi:10.1371/journal.pmed.1001439 23671407 PMC3646718

[14] Aichberger MC, Schouler-Ocak M, Mundt A, . Depression in middle-aged and older first generation migrants in Europe: results from the Survey of Health, Ageing and Retirement in Europe (SHARE). Eur Psychiatry. 2010;25(8):468-475. doi:10.1016/j.eurpsy.2009.11.009 20615669

[15] Heredia Montesinos A, Rapp MA, Temur-Erman S, Heinz A, Hegerl U, Schouler-Ocak M. The influence of stigma on depression, overall psychological distress, and somatization among female Turkish migrants. Eur Psychiatry. 2012;27(S2)(suppl 2):S22-S26. doi:10.1016/S0924-9338(12)75704-8 22863246

[16] Due C, Green E, Ziersch A. Psychological trauma and access to primary healthcare for people from refugee and asylum-seeker backgrounds: a mixed methods systematic review. Int J Ment Health Syst. 2020;14(1):71. doi:10.1186/s13033-020-00404-4 32944067 PMC7488556

[17] Lanzara R, Scipioni M, Conti C. A clinical-psychological perspective on somatization among immigrants: a systematic review. Front Psychol. 2019;9:2792. doi:10.3389/fpsyg.2018.02792 30705662 PMC6344401

[18] Iloson C, Möller A, Sundfeldt K, Bernhardsson S. Symptoms within somatization after sexual abuse among women: a scoping review. Acta Obstet Gynecol Scand. 2021;100(4):758-767. doi:10.1111/aogs.14084 33423277

[19] Nelson S, Baldwin N, Taylor J. Mental health problems and medically unexplained physical symptoms in adult survivors of childhood sexual abuse: an integrative literature review. J Psychiatr Ment Health Nurs. 2012;19(3):211-220. doi:10.1111/j.1365-2850.2011.01772.x 22070785

[20] Steffen-Klatt A, Fiess J, Beckh J, Schmidt R, Rockstroh B. The impact of adverse childhood experience on symptom severity in patients with functional neurological symptom disorder (FNSD). Ment Health Prev. 2019;13:169-175. doi:10.1016/j.mhp.2018.09.004

[21] Conrad D, Wilker S, Pfeiffer A, . Does trauma event type matter in the assessment of traumatic load? Eur J Psychotraumatol. 2017;8(1):1344079. doi:10.1080/20008198.2017.1344079 28804594 PMC5533143

[22] Arnetz BB, Broadbridge CL, Jamil H, . Specific trauma subtypes improve the predictive validity of the Harvard Trauma Questionnaire in Iraqi refugees. J Immigr Minor Health. 2014;16(6):1055-1061. doi:10.1007/s10903-014-9995-9 24549491 PMC4138298

[23] Breslau N, Chilcoat HD, Kessler RC, Davis GC. Previous exposure to trauma and PTSD effects of subsequent trauma: results from the Detroit Area Survey of Trauma. Am J Psychiatry. 1999;156(6):902-907. doi:10.1176/ajp.156.6.902 10360130

[24] Holbrook TL, Hoyt DB, Stein MB, Sieber WJ. Perceived threat to life predicts posttraumatic stress disorder after major trauma: risk factors and functional outcome. J Trauma. 2001;51(2):287-292. doi:10.1097/00005373-200108000-00010 11493786

[25] Stempel C, Sami N, Koga PM, Alemi Q, Smith V, Shirazi A. Gendered sources of distress and resilience among Afghan refugees in Northern California: a cross-sectional study. Int J Environ Res Public Health. 2016;14(1):25. doi:10.3390/ijerph14010025 28036054 PMC5295276

[26] Orang T, Ayoughi S, Moran JK, . The efficacy of narrative exposure therapy in a sample of Iranian women exposed to ongoing intimate partner violence: a randomized controlled trial. Clin Psychol Psychother. 2018;25(6):827-841. doi:10.1002/cpp.2318 30079583

[27] Keynejad RC, Hanlon C, Howard LM. Psychological interventions for common mental disorders in women experiencing intimate partner violence in low-income and middle-income countries: a systematic review and meta-analysis. Lancet Psychiatry. 2020;7(2):173-190. doi:10.1016/S2215-0366(19)30510-3 31981539 PMC7029417

[28] Breiman L. Random forests. Mach Learn. 2001;45(1):5-32. doi:10.1023/A:1010933404324

[29] Hothorn T, Hornik K, Strobl C, Zeileis A. party: A laboratory for recursive partytioning. February 1, 2014. Accessed June 12, 2023. https://cran.r-project.org/web/packages/party/vignettes/party.pdf

[30] Köbach A, Nandi C, Crombach A, Bambonyé M, Westner B, Elbert T. Violent offending promotes appetitive aggression rather than posttraumatic stress—a replication study with Burundian ex-combatants. Front Psychol. 2015;6:1755. doi:10.3389/fpsyg.2015.01755 26696913 PMC4672083

[31] Köbach A, Schaal S, Elbert T. Combat high or traumatic stress: violent offending is associated with appetitive aggression but not with symptoms of traumatic stress. Front Psychol. 2015;5:1518. doi:10.3389/fpsyg.2014.01518 25709586 PMC4285743

[32] World Medical Association. World Medical Association Declaration of Helsinki: ethical principles for medical research involving human subjects. JAMA. 2013;310(20):2191-2194. doi:10.1001/jama.2013.281053 24141714

[33] Mollica RF, Caspi-Yavin Y, Bollini P, Truong T, Tor S, Lavelle J. The Harvard Trauma Questionnaire: validating a cross-cultural instrument for measuring torture, trauma, and posttraumatic stress disorder in Indochinese refugees. J Nerv Ment Dis. 1992;180(2):111-116. doi:10.1097/00005053-199202000-00008 1737972

[34] Rasmussen A, Verkuilen J, Ho E, Fan Y. Posttraumatic stress disorder among refugees: measurement invariance of Harvard Trauma Questionnaire scores across global regions and response patterns. Psychol Assess. 2015;27(4):1160-1170. doi:10.1037/pas0000115 25894706 PMC4615261

[35] Peterman F, Brähler E. HSCL-25 Hopkins Symptom Checklist 25, Deutsche Version. Hogrefe; 2013.

[36] Franke GH. Symptom Checklist–90 Revised: Symptom Checklist, Deutsche Version. Beltz Test; 2002.

[37] Strobl C, Boulesteix AL, Zeileis A, Hothorn T. Bias in random forest variable importance measures: illustrations, sources and a solution. BMC Bioinformatics. 2007;8(1):25. doi:10.1186/1471-2105-8-25 17254353 PMC1796903

[38] Schalinski I, Teicher MH. Type and timing of childhood maltreatment and severity of shutdown dissociation in patients with schizophrenia spectrum disorder. PLoS One. 2015;10(5):e0127151. doi:10.1371/journal.pone.0127151 25992568 PMC4438058

[39] Schalinski I, Teicher MH, Rockstroh B. Early neglect is a key determinant of adult hair cortisol concentration and is associated with increased vulnerability to trauma in a transdiagnostic sample. Psychoneuroendocrinology. 2019;108:35-42. doi:10.1016/j.psyneuen.2019.06.007 31226659

[40] Tingley D, Yamamoto T, Hirose K, Keele L, Imai K. mediation: R package for causal mediation analysis. J Stat Softw. 2014;59(5):1-38. doi:10.18637/jss.v059.i0526917999

[41] Haldane J, Nickerson A. The impact of interpersonal and noninterpersonal trauma on psychological symptoms in refugees: the moderating role of gender and trauma type. J Trauma Stress. 2016;29(5):457-465. doi:10.1002/jts.22132 27603167

[42] Catani C. War at home: a review of the relationship between war trauma and family violence [in German]. Verhaltenstherapie. 2010;20(1):19-27. doi:10.1159/000261994

[43] Goessmann K, Ibrahim H, Saupe LB, Ismail AA, Neuner F. The contribution of mental health and gender attitudes to intimate partner violence in the context of war and displacement: evidence from a multi-informant couple survey in Iraq. Soc Sci Med. 2019;237:112457. doi:10.1016/j.socscimed.2019.112457 31387009

[44] Wachter K, Horn R, Friis E, . Drivers of intimate partner violence against women in three refugee camps. Violence Against Women. 2018;24(3):286-306. doi:10.1177/1077801216689163 29332516

[45] Spangaro J, Toole-Anstey C, MacPhail CL, Rambaldini-Gooding DC, Keevers L, Garcia-Moreno C. The impact of interventions to reduce risk and incidence of intimate partner violence and sexual violence in conflict and post-conflict states and other humanitarian crises in low and middle income countries: a systematic review. Confl Health. 2021;15(1):86. doi:10.1186/s13031-021-00417-x 34819111 PMC8611888

[46] Schouler-Ocak M. Mental health care for immigrants in Germany [in German]. Nervenarzt. 2015;86(11):1320-1325. doi:10.1007/s00115-015-4333-6 26385118

[47] Sammut D, Kuruppu J, Hegarty K, Bradbury-Jones C. Which violence against women educational strategies are effective for prequalifying health-care students? a systematic review. Trauma Violence Abuse. 2021;22(2):339-358. doi:10.1177/1524838019843198 31122182

[48] Zaher E, Keogh K, Ratnapalan S. Effect of domestic violence training: systematic review of randomized controlled trials. Can Fam Physician. 2014;60(7):618-624, e340-e347. 25022633 PMC4096259

