# Error in Coauthor’s First Name

**DOI:** 10.1001/jamanetworkopen.2023.33724

**Published:** 2023-09-01

In the Original Investigation titled “Traumatic Life Events and Association With Depression, Anxiety, and Somatization Symptoms in Female Refugees,”^1^ published July 20, 2023, coauthor Kneginja Richter’s first name was misspelled. This article has been corrected.^1^
