# Supplementary material for: Traumatic Life Events and Association With Depression, Anxiety, and Somatization Symptoms in Female Refugees
Source: JAMA Netw Open. 2023 Jul 20;6(7):e2324511. doi: 10.1001/jamanetworkopen.2023.24511 (PMC10359962; doi:10.1001/jamanetworkopen.2023.24511)
Supplement: Supplement. — Data Sharing Statement [file jamanetwopen-e2324511-s001.pdf]

Data Sharing Statement

Moran. Traumatic Life Events and Association With Depression, Anxiety, and Somatization Symptoms in Female Refugees. *JAMA Netw Open*. Published July 20, 2023.
doi:10.1001/jamanetworkopen.2023.24511

Data
Data available: No

Additional Information
Explanation for why data not available: The original consent forms for our participants did not specify permission for open access databases, so we cannot make the individual subject data available. We can however make the original analysis scripts available with a simulated dataset.
